# Uterine compression suture is an effective mode of treatment of postpartum haemorrhage

**DOI:** 10.12669/pjms.36.2.1072

**Published:** 2020

**Authors:** Hongmei Jiang, Li Wang, Jing Liang

**Affiliations:** 1 Hongmei Jiang, Binzhou People’s Hospital, Shandong, 256600, China; 2 Li Wang, Binzhou People’s Hospital, Shandong, 256600, China; 3 Jing Liang, Binzhou People’s Hospital, Shandong, 256600, China

**Keywords:** Uterine compression suture, Cesarean section, Postpartum hemorrhage

## Abstract

**Objective:**

To compare the effects of uterine compression suture versus conventional mode of treatment for the management of postpartum haemorrhage after caesarean section.

**Methods:**

This study enrolled 84 women with postpartum hemorrhage who were admitted to Binzhou People’s Hospital from August 2017 to October 2018 as the research subjects. They were divided into a control group and an observation group according to random number table method, 42 each group. The patients in the control group were treated by conventional treatment, while those in the observation group were treated by uterine compression suture. The hemorrhage, hemostasis, postoperative recovery and frequency of adverse reactions were compared between the two groups.

**Results:**

The amount of bleeding in the observation group was less than that in the control group, and the bleeding time was shorter than that in the control group; the differences had statistical significance (P<0.05). The success rate of hemostasis in the observation group was significantly higher than that in the control group, and the ineffective rate of hemostasis was significantly lower than that in the control group (P<0.05); the differences were statistically significant. The cleaning time of lochia, the recovery time of uterus and the recovery time of menstruation in the observation group were significantly shorter than that in the control group (P<0.05); the differences between the two groups were statistically significant (P<0.05). The frequency of adverse reactions in the observation group was significantly lower than that in the control group (P<0.05), and the difference was statistically significant (P<0.05).

**Conclusion:**

Uterine compression suture is effective for postpartum hemorrhage of cesarean section, which can effectively reduce postpartum hemorrhage, shorten postpartum hemorrhage time and accelerate the recovery. It is safe and worth clinical promotion.

## INTRODUCTION

Postpartum hemorrhage is a very common serious complication of puerpera during delivery. It refers to the situation that the amount of bleeding in vaginal delivery exceeds 500 mL and that in cesarean section exceeds 1000 mL within 24 hours after primary delivery. If postpartum hemorrhage is not handled promptly and properly, it will cause short-term or long-term complications, which will lead to maternal death.^1^-^3^ It has been reported that as many as 3% of the puerpera died of massive postpartum hemorrhage, which brought great pain to the puerpera and their families.^4^

In recent years, with the increasing rate of cesarean section, the proportion of puerpera with postpartum hemorrhage after cesarean section has gradually increased. It is very important to treat puerpera with postpartum hemorrhage after cesarean section as early as possible and effectively.^5^ At present, uterine artery ascending branch ligation, intrauterine gauze tamponade and arterial embolization are the main methods for stopping bleeding.^6^,^7^ Uterine gauze tamponade is easy to operate, but there is a risk of occult bleeding and delayed bleeding. Arterial embolization treatment requires high equipment and doctors’ technical requirements; hence it is not suitable for promotion. Hysterectomy can effectively control postpartum hemorrhage, but after losing the uterus as an endocrine organ, the ovarian secretory function of puerpera declines and they cannot be pregnant, which will produce negative impact on life quality; therefore it is not a conventional treatment method.^8^,^9^ Therefore, it is very important to find an effective way which is safe to control the bleeding after cesarean section. In recent years, studies show that uterine compression suture prevents postpartum hemorrhage by compressing and binding the arcuate vessels of uterine wall, slowing down and reducing blood flow, and promoting local tissue to form thrombosis to achieve effective hemostasis, and it has simple operation and rapid hemostatic effect.^10^,^11^ The hemostasis principle of uterine compression suture is as follows. Uterine compression suture makes the uterus in a state of oppression by binding and pressing, so as to better squeeze the blood vessels in the uterine wall, reduce the blood flow velocity of the uterus, and form local thrombus; in addition, under uterine compression, there will be ischemia in the myometrium of the uterus, which will stimulate the uterus to contract further and make the sinus close under pressure.^12^,^13^

The purpose of this study was to explore the clinical effect of uterine compression suture in the treatment of postpartum hemorrhage and provide a detailed reference for the promotion of uterine compression suture.

## METHODS

Eighty four puerpera with postpartum hemorrhage after cesarean section in Binzhou People’s Hospital from August 2017 to October 2018 were selected.

The sample size was obtained through:


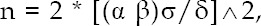


Where δ stands for the required degree of distinction, stands for the overall standard deviation or its estimated value s, and can be checked out from the row of degree of freedom v = ∞ -in the table of critical value of t.

All the patients had full-term pregnancy and had cesarean section indications.^14^ Those who had placental adhesion and placental implantation were excluded. According to the method of random number table; they were divided into the observation group and the control group. The age of puerpera in the observation group was 22-37 years old, and the average age was (30.6±3.1) years old; the gestational age was 37-42 weeks, and the average gestational week was (38.93±2.68) weeks; there were 20 cases of primipara and 22 cases of transpartum; as to the causes of bleeding, there were nine cases of macrosomia, 10 cases of twin pregnancy, eight cases of placenta previa, 2 cases of gestational hypertension, two cases of scarred uterus, and 11 cases of two or more causes. The age of puerperal in the control group was 21-38 years old, with an average age of (30.9±3.2) years; the gestational age was 37-41 weeks, with an average gestational age of (38.92±2.69) weeks; there were 21 were primipara and 21 were transpartum; as to the causes of bleeding, there were 10 cases of macrosomia, 12 cases of twin pregnancy, eight cases of placenta previa, 2 cases of gestational hypertension, one case of scarred uterus, and nine cases of two or more causes. There was no significant difference between the two groups in terms of age, gestational age and bleeding causes (P>0.05). This study has been reviewed and approved by the hospital ethics committee, and the puerpera and their family members voluntarily signed the informed consent.. (129 dated Feb. 26, 2019)

### Methods:

All the preoperative examinations were completed in both groups. After combined spinal-epidural anesthesia, cesarean section of lower uterus was performed in both groups. The control group received conventional hemostasis methods. After delivery, the amount of postpartum hemorrhage was estimated. When the amount of postpartum hemorrhage reached 300 ml, great attention was paid to find out the specific causes of bleeding, and then targeted treatment was carried out. If the amount of vaginal bleeding after placenta delivery was large, it was considered whether it was caused by residual placental tissue or uterine contraction weakness. If the uterine contraction was week, uterine gauze filling, oxytocin or uterine massage were used. If the bleeding was caused by tissue residue or placental retention, the placental tissue was cleaned timely.

The observation group received the improved B-Lynch uterine compression suture. Firstly, the uterine cavity was emptied and sutured using No. 1 absorbable suture. The needle was inserted from the site which was 2-3 cm away from the inferior margin of the right uterine incision and 3 cm away from the medial margin of the uterine. The absorbable suture was pulled to the fundus and then vertically towards the posterior wall of the uterine from the site which was 3-4 cm away from the right corner, in that process, the uterine seromuscular layer at the anterior and posterior uterine wall and fundus were sutured three stitches, and the suture was fixed to prevent slip. Then the suture of the posterior wall was inserted at the position which was relative to the anterior wall, withdrawn from the left posterior wall of the uterine, and bypassed the fundus to the anterior wall using the same suture method. During suture, the assistant compressed the uterine body with his hands and tied a knot below the incision at the lower uterine segment after the suture was tightened. After the checking of suture and effective hemostasis, the uterine incision was sutured, and the abdominal cavity was sutured layer by layer.

### Observation indicators

1. The amount of postpartum hemorrhage and the time of bleeding were compared between the two groups and recorded in detail. The volume method was used to measure the amount of bleeding. Amniotic fluid in the negative pressure bottle was first recorded after absorption of amniotic fluid during cesarean section, and then the amount of bleeding was collected with a suction device. The amount of blood in the negative pressure bottle was recorded at the end of operation; the bleeding amount could be obtained by subtracting amniotic fluid volume from the amount of blood in the negative pressure bottle at the end of operation. 10 mL of blood was recorded when no blood dropped from the gauze in a size of 10 cm×10 cm which was saturated.
2. The hemostatic effect of two groups of patients were compared and analyzed. The specific evaluation criteria are as follows. Hemostasis was considered as ineffective if vaginal bleeding was not effectively improved, the amount of vaginal bleeding was significantly higher than that of normal amount of lochia, and even there was hemorrhagic shock. Bleeding was determined as reduced if the amount of vaginal bleeding was significantly reduced compared to before treatment, but bleeding was not completely stopped and needed to be stopped by drugs. Hemostasis was evaluated as effective if the amount of vaginal bleeding was significantly reduced and the bleeding amount was equivalent to the normal amount of lochia.
3. The recovery of postpartum women in the two groups was observed, including cleaning time of lochia, uterus recovery time and menstrual recovery time.
4. The occurrence of adverse reactions such as uterine incision dehiscence and puerperal infection in the two groups were observed.

### Statistical analysis

SPSS 21.0 was used for statistical analysis and processing of all data. The results of measurement data were expressed by Mean±SD; the pairwise comparison adopted *t* test. The results of counting data were expressed by percentage and processed by X^2^ test. Difference was considered as statistically significant if P < 0.05.

## RESULTS

The amount of bleeding during operation and 2 hours after operation in the observation group was less than that in the control group, and the bleeding time after operation was shorter than that in the control group. The differences were statistically significant (P<0.05, Table-I).

**Table-I:** Hemorrhage between two groups (Mean±SD).

Group	Observation group	Control group	t	P
Intraoperative bleeding volume (mL)	894.37±134.25	1123.75±204.17	7.152	<0.05
Hemorrhage volume 2 hours after operation (mL)	33.58±6.72	49.36±7.21	5.543	<0.05
Postoperative bleeding time (h)	8.23±1.33	12.35±3.45	3.167	<0.05

The success rate of hemostasis in the observation group was significantly higher than that in the control group, and the ineffective rate of hemostasis was significantly lower than that in the control group; the differences were statistically significant (P<0.05, Table-II).

**Table-II:** Hemostatic effect between two groups after treatment (%).

Group	Observation group	Control group	t	P
Success rate of hemostasis	39(92.86)	35(83.33)	6.668	<0.05
Bleeding reduction rate	3(7.14)	4(9.52)	0.384	>0.05
Ineffective rate of hemostasis	0(0)	3(7.14)	5.457	<0.05

The cleaning time of lochia, recovery time of uterus and menstrual recovery time in the observation group were significantly shorter than those in the control group (P<0.05) and the differences were statistically significant (Table-III).

**Table-III:** Postoperative recovery between two groups (Mean±SD).

Group	Observation group	Control group	t	P
Cleaning time of lochia (week)	5.23±1.46	9.68±1.85	7.146	<0.05
Recovery time of uterus (d)	46.16±3.45	55.36±3.59	6.738	<0.05
Menstrual recovery time (month)	4.59±1.37	8.78±1.65	2.471	<0.05

There were no cases of hysterectomy due to secondary bleeding after operation in both groups. The total incidences of adverse reactions was 4.76% (2/42) in the observation group, including one case of puerperal infection and one case of obstruction. The total incidence of adverse reactions was 19.05% (8/42) in the control group, including one case of adhesion, five cases of puerperal infection and 2 cases of obstruction. The total incidence of adverse reactions in the observation group was significantly lower than that in the control group (X^2^=4.672, P<0.05).

## DISCUSSION

Currently, the treatment methods for postpartum hemorrhage include uterine massage, oxytocin therapy and uterine gauze tamping, etc. When the hemostatic effect of the above methods is not significant, uterine compression suture can be used. Uterine compression suture is a safe and effective method for hemostasis. A relevant study has shown that uterine compression suture has a significantly shorter operative time and a significantly lower amount of blood loss compared with the method of uterine wall compression with gauze packing.^15^

In this study, the effects of uterine compression suture and conventional treatment were compared. The results showed that the amount of bleeding during operation and after operation in the observation group was significantly lower than that in the control group, which indicated that the suture in uterine compression suture was only performed in muscular layer and the characteristics of hemostasis by compression determined that the amount of bleeding during and after operation was low, and the findings were similar to the previous research results.^16^,^17^ In addition, the results also showed that the success rate of hemostasis in the observation group was significantly higher than that in the control group, and the ineffective rate of hemostasis was significantly lower than that in the control group (P<0.05). Those results fully demonstrated that uterine compression suture could achieve remarkable results in the treatment of postpartum hemorrhage, help patients stop bleeding quickly, and improve the success rate and effective rate of hemostasis and it was safe and effective.

In previous studies, most of the literatures focused on the hemostasis effect of various surgical methods in the treatment of postpartum hemorrhage during cesarean section and did not investigate the postpartum recovery and complications.^18^,^19^ However, this study further carried out a long-term follow up on the patients. The results showed that the cleaning time of lochia, recovery time of uterus and menstrual recovery time of the observation group were significantly shorter than those of the control group, which fully showed that uterine compression suture could greatly shorten the hemostasis time, lochia cleaning time, recovery time of uterine and menstrual recovery time of patients, and the incidence of complications in the observation group was significantly lower than that in the control group. Uterine compression suture was not easy to cause intestinal obstruction and intussusceptions as the suture was seldom slipped, and moreover the discharge of lochia was seldom obstructed as the suture was not tight, which were similar to the research results of Jia.^20^

## CONCLUSION

Uterine compression suture is significantly effective in treating postpartum hemorrhage during cesarean section. Uterine compression suture is simple in operation so that clinical physicians can easily acquire proficiency in it and the bleeding can be rapidly stopped, which can greatly save blood resource. Moreover it can significantly improve the quality of life of puerpera, showing a great superiority. Therefore it is worth promotion. However, the number of cases included in this study was small, and they all come from single center, which limited the study. Long-term follow-up results of more cases are needed in the future.

### Authors’ Contribution:

**HMJ** & **LW:** Study design, data collection and analysis.

**LW & JL:** Manuscript preparation, drafting and revising.

**HMJ:** Review and final approval of manuscript, is responsible for integrity of research.
